# A rare case of *Cysticercus tenuicollis* infection in a neonate lamb: Evidence of prenatal transmission

**DOI:** 10.1002/vms3.1341

**Published:** 2023-12-20

**Authors:** Peyman Dehghan Rahimabadi, Javad Abbasi, Alireza Shaghayegh, Nadia Taefi Nasrabadi, Diba Golchin, Yasaman Kavakebi Asar, Iradj Ashrafi Tamai, Amin Anoushepour

**Affiliations:** ^1^ Department of Clinical Sciences Karaj Branch Islamic Azad University Karaj Iran; ^2^ Department of Animal and Poultry Health and Nutrition Faculty of Veterinary Medicine University of Tehran Tehran Iran; ^3^ Institute of Biomedical Research University of Tehran Tehran Iran; ^4^ Department of Parasitology Karaj Branch Islamic Azad University Karaj Iran; ^5^ Department of Pathobiology Faculty of Veterinary Medicine University of Tehran Tehran Iran; ^6^ Student of Veterinary Medicine Karaj Branch Islamic Azad University Karaj Iran; ^7^ Department of Microbiology and Immunology Faculty of Veterinary Medicine University of Tehran Tehran Iran

**Keywords:** *Cysticercus tenuicolli*s, histopathology, intrauterine infestation, pancreas, PCR

## Abstract

Cysticercosis develops in lambs following a *Cysticercus tenuicollis* infestation, which is the larval stage of *Taenia hydatigena*. A 7‐day‐old lamb was examined for depression, anorexia, fever (40.5°C), congested mucus membranes, reluctance to move, and a hunched back. Upon necropsy, congestion was noted in the intestines and brain, and the heart had a loose consistency. Soft and pulpy kidneys were evident coupled with watery intestinal contents. Epsilon toxin (*Clostridium perfringens* type D toxin) was detected using enzyme‐linked immunosorbent assay. A transparent cystic structure was incidentally found attached to the pancreas, within which a scolex was well demonstrated upon histopathology. Chronic active peritonitis was diagnosed at the cyst attachment site. *C. tenuicollis* was confirmed by polymerase chain reaction and genome sequencing. This report describes prenatal transmission of *C. tenuicollis* in the present lamb, although this condition is quite rare.

## Graphical abstract

*Cysticercus tenuicollis*, the larval stage of *Taenia hydatigena*, can form bladder cysts in various visceral organs in lambs. Although there have been previous reports of the parasite invading reproductive system of ewes as well as fetal membranes, to date, there has been no documentation of fetal visceral involvement. Here we describe a prenatal transmission of *C. tenuicollis* in a 7‐day‐old lamb.

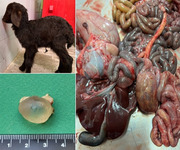

## 1 INTRODUCTION

Cysticercosis is a chronic disease caused by *Cysticercus tenuicollis*, the larval stage (metacestode) of *Taenia hydatigena* tapeworm (Al‐Mayali, 2005; Sgroi et al., 2020), which can lead to both acute and chronic forms of cysticercosis in sheep, goats, cattle, pigs and humans (Corda et al., 2020; Haddawee et al., 2018).

*T. hydatigena* lives in the intestine of definitive hosts such as dogs, cats, foxes, wolves, lynx, jackals, raccoons, and bears (Corda et al., 2020). The proglottids or eggs are excreted in the faeces and can be transmitted to the intermediate hosts when they are ingested along with plants or grasses. The eggs further hatch to oncospheres in the small intestine and reach the portal circulation as they penetrate the intestinal wall. Subsequently, these oncospheres reach the liver (Al‐Hamzawi & Al‐Mayali, 2020; Corda et al., 2020; Haddawee et al., 2018). They can be found in the liver parenchyma within 7–10 days post‐infection (Corda et al., 2020). Most oncospheres leave the liver and enter the peritoneal cavity, where they develop into *C. tenuicollis* (Haddawee et al., 2018; Hama et al., 2018), that has a long neck and transparent wall (bladder cyst). The length and size of the cysts vary from one to several centimetres. They are usually found attached to various organs, including the omentum, mesentery, lungs, kidneys, spleen, brain, rumen, heart, gall bladder, diaphragm, urinary bladder, ovaries, uterus, uterine tubes, cervix, vagina, and sometimes on the liver surface, particularly in sheep and goats (Al‐Hamzawi & Al‐Mayali, 2020; Haddawee et al., 2018; Hama et al., 2018; Radfar et al., 2005; Sgroi et al., 2020). The definitive hosts become infected after consumption of the cysts present in the infected animal's offals. Then, after 51 days the worm becomes mature in the small intestine (Al‐Hamzawi & Al‐Mayali, 2020; Hama et al., 2018).

Diagnosis can be best achieved at the time of necropsy. Serological tests, such as ELISA and Western blot, have a low sensitivity in naturally occurring infections. Additionally, molecular diagnostic tests can be beneficial in confirmation of *C. tenuicollis* (Hama et al., 2018). The control the infection in the definitive hosts relies on a regular treatment of dogs with antiparasitic drugs (Niclosamide or Praziquantel), which is crucial to avoid dogs from accessing sheep and goat carcasses and offals (Mohammed, 2020).

The disease can cause economic losses, and its prevalence varies among poor and developing countries (Hama et al., 2018; Sgroi et al., 2020). Cysticercosis has been documented in old‐aged sheep and goats worldwide (Al‐Mayali, 2005; Abbas et al., 2021), but it is considered rare in young lambs (Al Salihi et al., 2016).

This study reports on a case of cysticercosis in a 7‐day‐old lamb, which is believed to have acquired a congenital infection.

## 2 CASE DESCRIPTION

### 2.1 Clinical presentation

**FIGURE 1:**
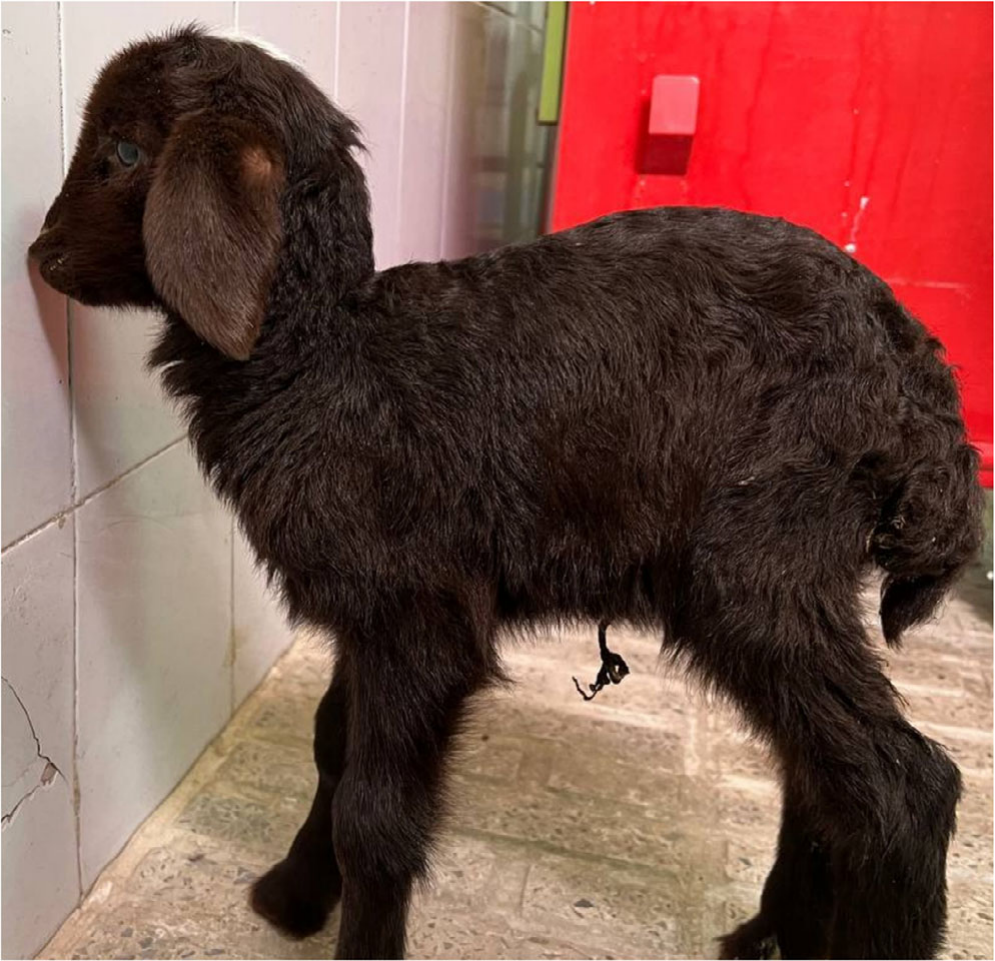
The index lamb with drooping ears and hunched back.

In March 2023, a 7‐day‐old mixed breed male lamb was presented to Veterinary Hospital, Nazarabad, Alborz, Iran with depression, anorexia, diarrhoea, fever (40.5°C), congested mucous membranes, reluctance to move, and a hunched back (Figure 1).

The patient's heart and respiratory rates were 100 beats/min and 35 breaths/min, respectively. The lamb became recumbent after 1 h and died in 2 h. Thus, necropsy was conducted.

According to the owner, ten 1–3‐week‐old lambs had recently died in his farm with similar clinical signs.

### 2.2 Necropsy

**FIGURE 2:**
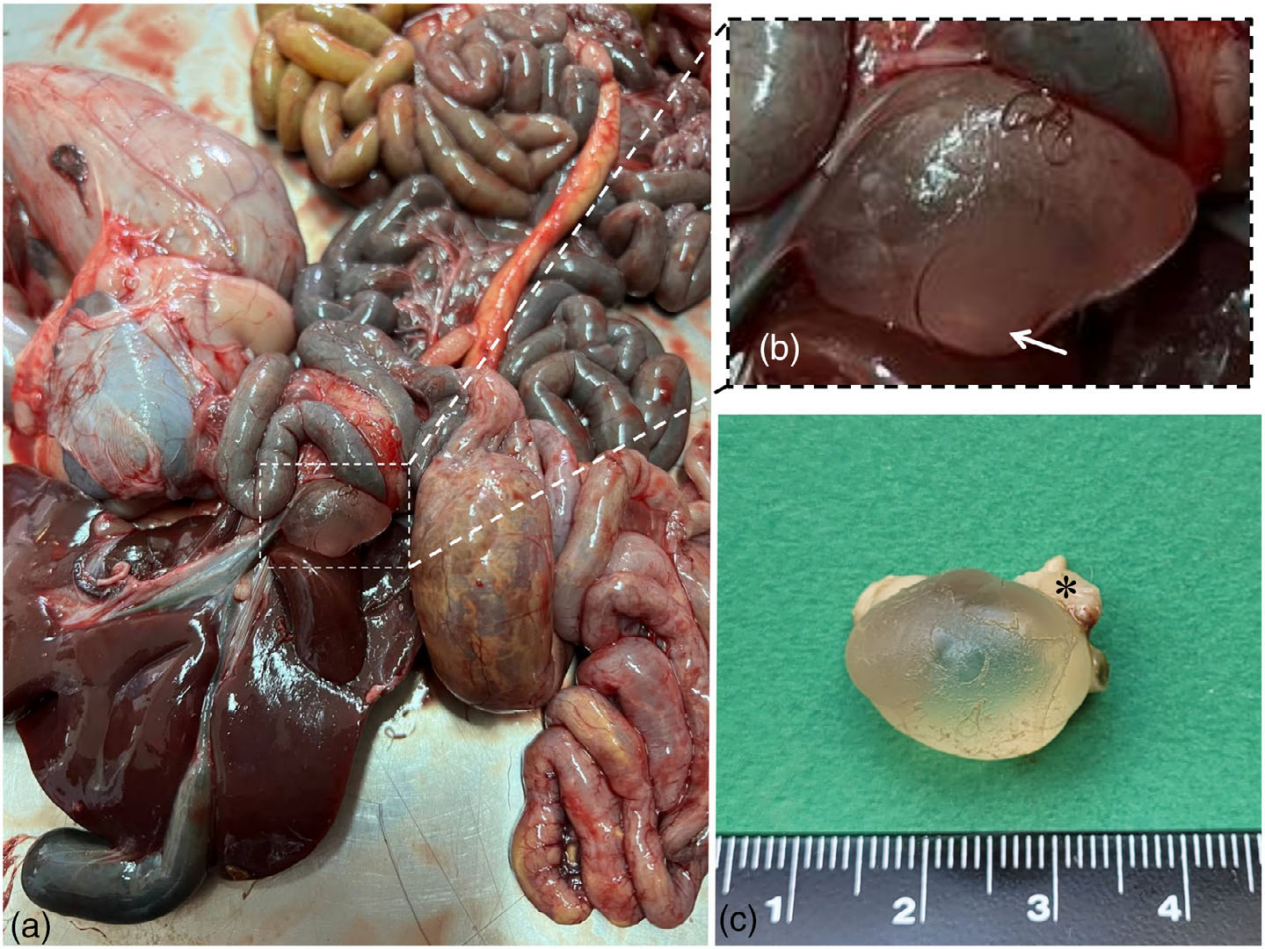
(A) The cyst is depicted adjacent to the liver. Note serosal hyperemia of the small intestine. (B) The presence of a single scolex within the translucent fluid‐filled cyst (arrow). (C) The resected and formalin‐fixed cyst is depicted with the associated pancreatic tissue (asterisk) measuring 1.8 × 1.5 × 1 cm^3^.

Necropsy revealed congestion of the intestines and brain, soft pulpy kidneys, loose consistency of the heart, and watery intestinal contents. A volume of 5 mL of the intestinal contents was referred to the laboratory to examine for the *Clostridium perfringens* type D toxin (epsilon toxin). A commercial ELISA kit (Bio‐X Diagnostics) was used that confirmed the presence of the suspected toxin. In the light of these findings, the lamb's death was attributed to enterotoxaemia. Interestingly, a thin‐walled cyst, measuring 1.8 × 1.5 × 1 cm^3^, was noted adjacent to the liver and attached to the pancreas (Figure 2A). A scolex was noted within the cyst (Figure 2B). No additional cysts were detected upon necropsy. The cyst and the associated pancreatic tissue (Figure 2C) were collected as well as samples of liver and were immersed in 10% neutral buffered formalin for histopathologic and molecular examinations.

### 2.3 Histopathological examination

**FIGURE 3:**
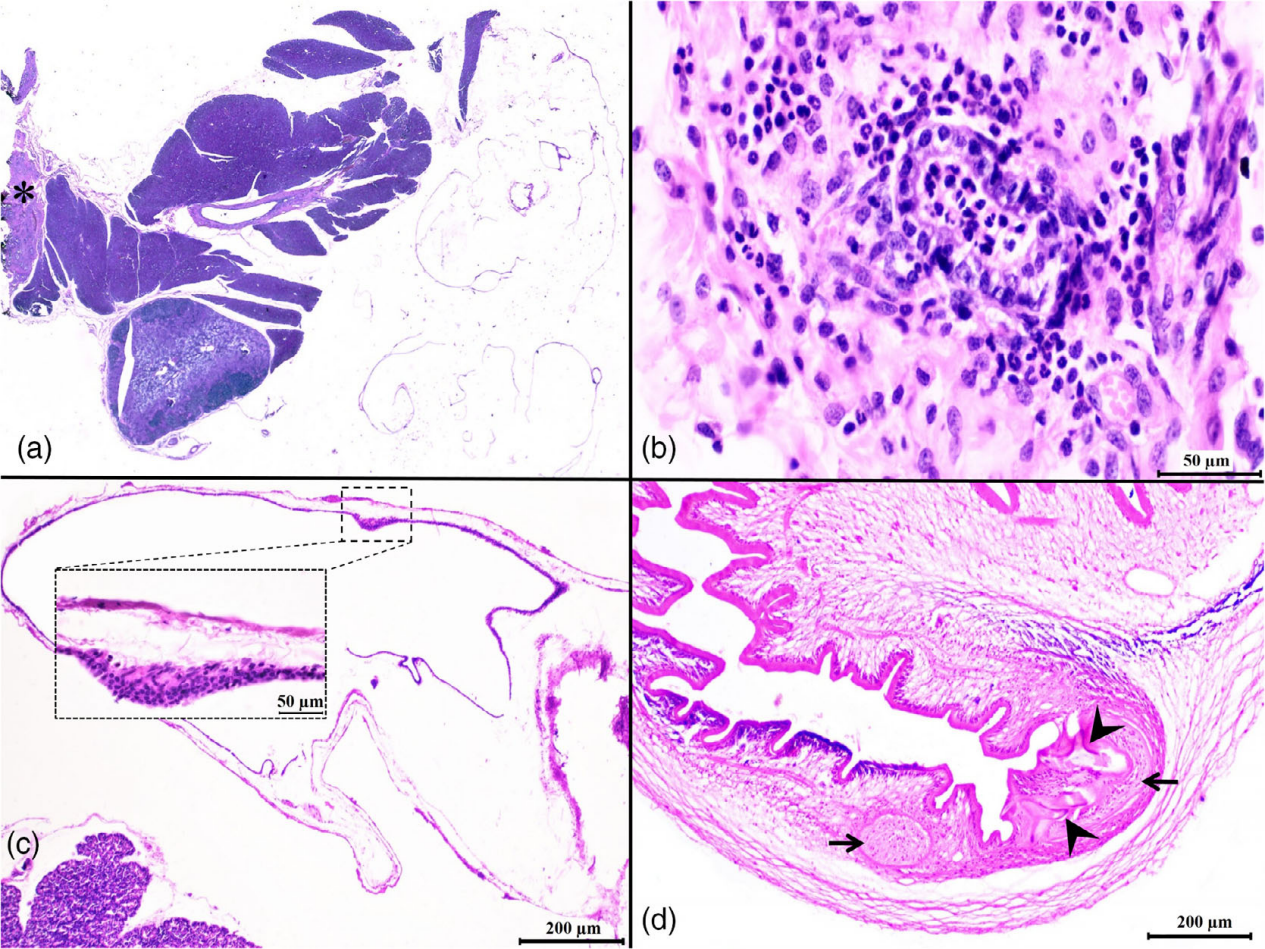
(A) Subgross photograph depicts the cyst attached to the pancreas coupled with a locally extensive neutrophilic peritonitis (asterisk) (haematoxylin and eosin [H&E]). (B) Higher magnification shows septic peritonitis at the periphery of the pancreas (H&E, 400×). (C) Microphotograph of the cyst wall reveals its association with the pancreatic parenchyma (H&E, 100×). Higher magnification shows the cellular elements of the inner layer of the cyst wall. (H&E, 400×). (D) Hooklets (arrowheads) and suckers (arrows) of the armed scolex residing inside the cyst are depicted (H&E, 100×).

Specimens were processed according to the standard histological techniques, embedded in paraffin, sectioned at 5 μm thickness, and stained by haematoxylin and eosin (Carson & Cappellano, 2015). The slides were then reviewed by a light microscope (Olympus, CX33). The scolex was isolated and processed separately for histologic evaluation. Upon histopathology, a thin‐walled cyst was noted attached to the pancreatic surface, whereas the pancreas was otherwise normal (Figure 3A,C). Chronic active peritonitis at the pancreatic attachment site and mild neutrophilic lymphadenitis were other remarkable histopathologic findings (Figure 3B). The armed scolex was visualized. The microanatomy of the scolex and its hooklets and suckers were visible (Figure 3D). The liver sections appeared normal upon gross and microscopic inspection.

### 2.4 Polymerase chain reaction (PCR) and nucleotide sequence analysis

**FIGURE 4:**
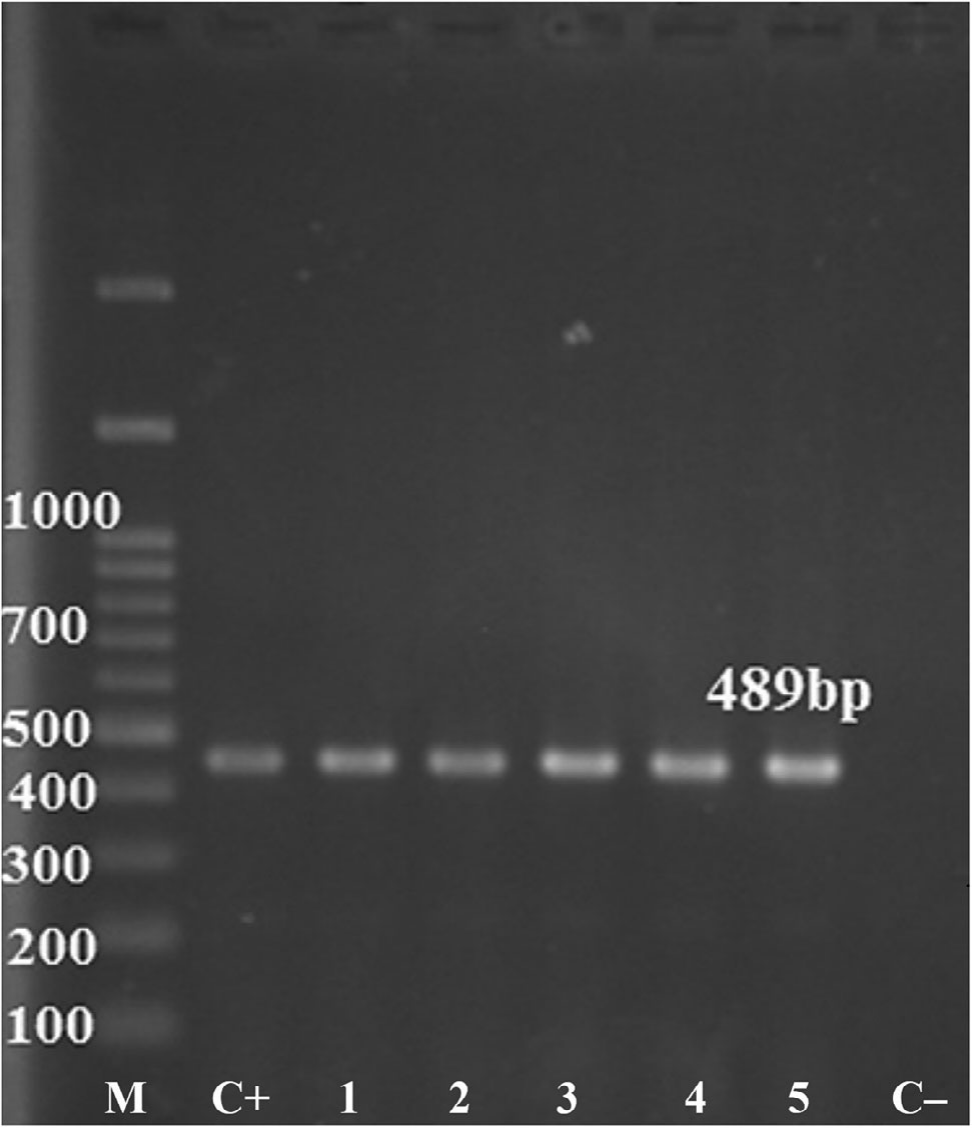
Amplified PCR products of 489 bp in size from *Cysticercus tenuicollis* run on agarose gel. Marker 100 bp: M; positive control: C+; cyst fluid: 1 and 2; cyst wall: 3, 4 and 5; negative control: C−.

DNA was extracted from the cyst fluid and wall, using a commercial kit (SinaPure DNA, SinaClon) according to the manufacturer's instructions. A pair of primers *JB3* (5'‐TTTTTTGGGCATCCTGAGGTTTAT‐3', as the forward primer) and *JB4.5* (5'‐TAAAGAAAGAACATAATGAAAATG‐3', as the revers primer) was used to amplify the 489 bp fragment of the cytochrome *c* oxidase subunit I (COX1) gene (Bowles et al., 1992). Polymerase chain reaction (PCR) was conducted in 25 μL reaction mixtures containing 2 mM of MgCl_2_ (Fermentas), 0.2 mM of each dNTP (Fermentas), 2.5 μL of 10× PCR buffer, 2 U of Taq DNA Polymerase (Fermentas), 10 pmol of each primer (Bioneer) and 2 μL of DNA template. The reactions were carried out in a thermocycler (TC‐512 Techne) as follows; an initial denaturation at 95°C for 10 min, then 35 cycles of 95°C for 30 s, 55°C for 30 s, 72°C for 30 s, and a final extension at 72°C for 7 min. The amplification products (5 μL) were resolved by electrophoresis. The 1% agarose gel was then stained using 1 μg/mL ethidium bromide (CinnaGen) and visualized by a UV transilluminator (Bio‐Rad) (Figure 4).

The PCR product was extracted, purified and sequenced (Bowles et al., 1992) at Codon Genetic Group, Iran. The partial nucleotide sequence of mitochondrial COX1 gene was submitted to the GenBank (PeyVad2023; accession number: OR209738), which was verified as *T. hydatigena*.

**FIGURE 5:**
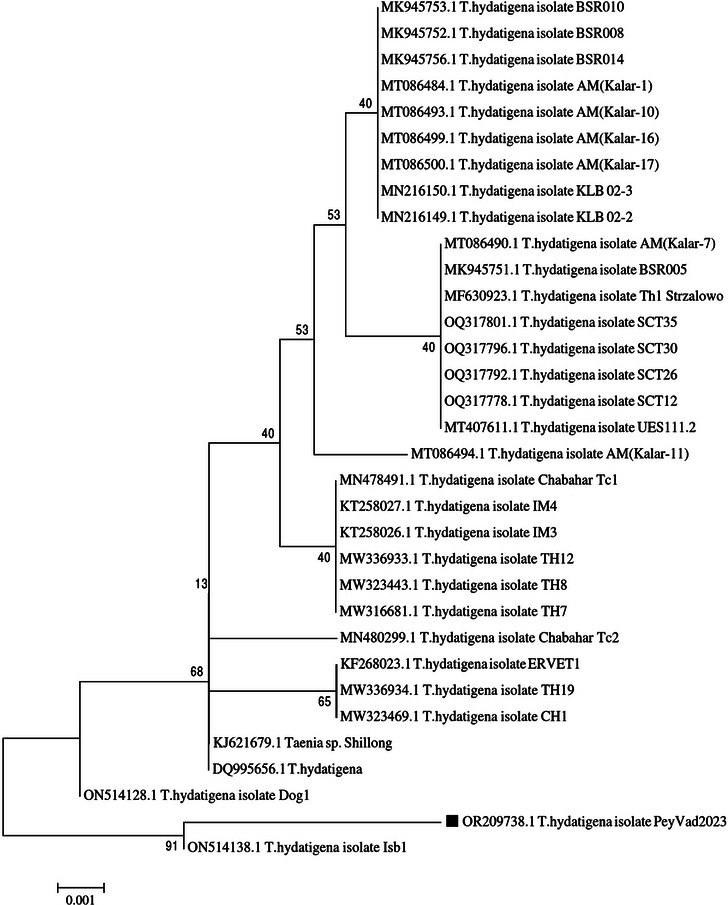
Phylogenetic analysis of *Taenia hydatigena* sequences, based on COX1 gene in neighbour‐joining tree.

For drawing of the phylogenetic tree, bootstrap test (1000 replicates) for neighbour‐joining and maximum composite likelihood methods and other settings were obtained using the default values in MEGA7 software. Moreover, 32 sequences obtained from GenBank were used for comparisons (Figure 5).

## 3 DISCUSSION

*C. tenuicollis* infection is widely reported from herbivores and is more prevalent in countries with lower sanitary measures (Payan‐Carreira et al., 2008). Given the endemic nature of *T. hydatigena* in dogs and wild carnivores in Iran, the disease is frequently diagnosed in sheep and goats (Nourani et al., 2010; Radfar et al., 2005). Cysticercosis often occurs in the first months of age in lambs but is only diagnosed in 4–11 months, after the cysts are developed (Corda et al., 2020). After the egg enters the intestinal tract, it hatches and the oncosphere reaches the liver. At this point, it takes 4 weeks for the larva to migrate through the liver and reach to the hepatic surfaces and other visceral organs. Four additional weeks are required for the oncosphere to develop into a cyst in which the metacestode resides (Haddawee et al., 2018). Thus, a minimum of 8–10 weeks is required for the development of *C. tenuicollis* in the viscera of a lamb. That is while the present lamb was only 7‐day old, this suggests a fetal transfer of *C. tenuicollis*. Although *C. tenuicollis* is usually found on the omentum and peritoneum (due to their vast surface area) (Corda et al., 2020; Haddawee et al., 2018; Mohammed, 2020), it has also been reported in various visceral organs (Mohammed, 2020; Payan‐Carreira et al., 2008). To the authors’ knowledge, to date, these cysts have not been reported to develop over pancreatic surfaces. Payan‐Carreira et al. (2008) have reported *C. tenuicollis* in the ovaries, uterine tube, uterus, cervix, and vagina of a pregnant ewe in addition to the fetal membranes, whereas upon necropsy, visceral involvement was not observed in the fetus. They also suggested that the oncosphere can migrate to the developing fetus in pregnant animals. Smith et al. (1999) have detected *C. tenuicollis* on the uterine tube and broad ligament in a ewe. Al Salihi et al. (2016) have described the cyst over the pregnant uterine body of a goat and the fetal body. They have stated that the presence of *C. tenuicollis* increases the chance of the oncosphere reaching the fetus. In older animals, the period of *C. tenuicollis* exposure during feeding is prolonged. On the other hand, the longer life span of ewes confronts them with more physiologic stress such as pregnancy (Dey et al., 2022). Previous studies have stated that the oncosphere may reach the cotyledon, placental wall, allantoic membrane vascular network, and gradually the allantoic cavity through small‐caliber capillaries (Al Salihi et al., 2016; Payan‐Carreira et al., 2008). This means that the fetus can ingest the free‐floating oncosphere with the amniotic fluid as a route of entry. Cestode oncospheres can escape from the immunological reactions by a modulation of the immune response (immunomodulation) (Al Salihi et al., 2016). Moreover, the poor development of the immune system in the fetus facilitates oncosphere migration and development of the parasitic cyst (Payan‐Carreira et al., 2008).

In the migration phase, haemorrhage within the hepatic parenchyma and surface (haemorrhagic streaks), infiltration of inflammatory cells and fibrosis around the cysticerci are the most significant histopathologic findings (Al‐Mayali, 2005; Blazek et al., 1985; Nourani et al., 2010; Pathak et al., 1982). These are indicative lesions of *C. tenuicollis*, especially in endemic regions (Corda et al., 2020). That is while in the present lamb, the liver was grossly intact. This can be due to the migration of only one larva into the liver, imposing minimal lesions which were missed upon sampling or these mild lesions may have resolved over time. Al Salihi et al. (2016) have reported haemorrhage, fibrin deposition, and degeneration of the uterine tissue in the attachment site of *C. tenuicollis* (Al Salihi et al., 2016). In the index case, focal peritonitis was diagnosed adjacent to the pancreas, which is contrary to the findings described by Payan‐Carreira et al. (2008) where no active inflammation was noticed in the cotyledons and uterine wall. However, there are reports describing peritonitis caused by free‐floating larvae (Darzi et al., 2002). Blazek et al. (1985) have reported serofibrinous peritonitis on day 10, and exudative peritonitis on day 10–14 post‐infection in pigs.

The combination of necropsy and molecular characterization (DNA identification methods) is essential for the understanding of the nature, species, strain, and origin of cysts (Hama et al., 2018; Mohammed, 2020). As in the present case, the mitochondrial cytochrome COX1 gene is one of the most popular mt‐DNA genes that are used for studying the phylogeny of helminth parasites (Dey et al., 2022; McManus, 2006; Mohammed, 2020; Rostami et al., 2015; Raissi et al., 2021). Moreover, the alignment and blast of the present sequence showed 99.18% and 99.45% similarity to ON514138 and ON514128, respectively, deposited to GenBank from Pakistan.

As the population of dogs and other wild carnivores is not controlled in Iran, the occurrence of cysticercosis in farm animals is expectable. In the present report PCR analysis of the cyst wall and fluid confirmed *C. tenuicollis*. Additionally, the scolex was well demonstrated upon histopathology. This report describes prenatal transmission of *C. tenuicollis* in the lamb, although this condition is quite rare.

## AUTHOR CONTRIBUTIONS

*Investigation and writing – original draft*: Peyman Dehghan Rahimabadi, Diba Golchin, Yasaman Kavakebi Asar and Iradj Ashrafi Tamai. *Reviewer of literature*: Javad Abbasi, Alireza Shaghayegh, Nadia Taefi Nasrabadi, Amin Anoushepour. *Writing – review and editing*: Peyman Dehghan Rahimabadi and Diba Golchin.

## AKNOWLEDGEMENTS

The authors would like to express their heartfelt gratitude to Mr. Raza Agha Ebrahim Samani, for his invaluable support and assistance.

## CONFLICT OF INTEREST STATEMENT

The authors declare that they have no conflicts of interest.

### ETHICS STATEMENT

The authors confirm that the ethical policies of the journal, as noted on the journal's author guidelines page, have been adhered to. No ethical approval was required as the study was conducted on the cadaver with the owner's consent.

### PEER REVIEW

The peer review history for this article is available at https://publons.com/publon/10.1002/vms3.1341

## Data Availability

Data sharing not applicable to this article as no datasets were generated or analysed during the current study.
